# Protein Binding Partners of Dysregulated miRNAs in Parkinson’s Disease Serum

**DOI:** 10.3390/cells10040791

**Published:** 2021-04-02

**Authors:** Wolfgang P. Ruf, Axel Freischmidt, Veselin Grozdanov, Valerie Roth, Sarah J. Brockmann, Brit Mollenhauer, Dorothea Martin, Bernhard Haslinger, Katrin Fundel-Clemens, Markus Otto, Christine von Arnim, Karlheinz Holzmann, Albert C. Ludolph, Jochen H. Weishaupt, Karin M. Danzer

**Affiliations:** 1 Department of Neurology, Ulm University, 89081 Ulm, Germany; wolfgang.ruf@uni-ulm.de (W.P.R.); axel.freischmidt@uni-ulm.de (A.F.); veselin.grozdanov@uni-ulm.de (V.G.); valerie.roth@christophorus-kliniken.de (V.R.); sarah.brockmann@uni-ulm.de (S.J.B.); markus.otto@uni-ulm.de (M.O.); Albert.Ludolph@rku.de (A.C.L.); 2 German Center for Neurodegenerative Diseases (DNZE), 89081 Ulm, Germany; 3 Department of Neurology, Universitätsmedizin Göttingen and Paracelsus-Elena-Klinik, 34128 Kassel, Germany; brit.mollenhauer@paracelsus-kliniken.de; 4 Department of Neurology, Technische Universität München, 80333 Munich, Germany; Dorothea.a.martin@gmail.com (D.M.); bernhard.haslinger@tum.de (B.H.); 5 Boehringer Ingelheim Pharma GmbH & Co. KG, Div. Research Department, 88400 Biberach, Germany; katrin.fundel-clemens@boehringer-ingelheim.com; 6 Department of Geriatrics, Göttingen University, 37075 Göttingen, Germany; christine.arnim@med.uni-goettingen.de; 7 Genomics-Core Facility, Center for Biomedical Research, University Hospital Ulm, 89081 Ulm, Germany; karlheinz.holzmann@uni-ulm.de; 8 Institute for Neurodegeneration, Universitätsmedizin Mannheim, 68167 Mannheim, Germany; Jochen.Weishaupt@medma.uni-heidelberg.de

**Keywords:** Parkinson’s disease, miRNA, serum

## Abstract

Accumulating evidence suggests that microRNAs (miRNAs) are a contributing factor to neurodegenerative diseases. Although altered miRNA profiles in serum or plasma have been reported for several neurodegenerative diseases, little is known about the interaction between dysregulated miRNAs and their protein binding partners. We found significant alterations of the miRNA abundance pattern in serum and in isolated serum-derived extracellular vesicles of Parkinson’s disease (PD) patients. The differential expression of miRNA in PD patients was more robust in serum than in isolated extracellular vesicles and could separate PD patients from healthy controls in an unsupervised approach to a high degree. We identified a novel protein interaction partner for the strongly dysregulated hsa-mir-4745-5p. Our study provides further evidence for the involvement of miRNAs and HNF4a in PD. The demonstration that miRNA-protein binding might mediate the pathologic effects of HNF4a both by direct binding to it and by binding to proteins regulated by it suggests a complex role for miRNAs in pathology beyond the dysregulation of transcription.

## 1. Introduction

Parkinson’s disease (PD), a complex multi-factorial disease, is believed to result from the interplay of genetic and environmental factors. Genetic predisposition and environmental factors, such as lifestyle and environmental exposures, result in long-lasting changes in gene expression. These changes, termed epigenetic modulation, are mediated by DNA methylation, chromatin reorganization, and small non-coding RNAs. Small non-coding RNAs, such as miRNAs, have recently emerged as a potential factor in PD and other neurodegenerative diseases. MiRNAs regulate gene expression at the posttranscriptional level [1], contributing to a high degree of transcriptomic plasticity. Since miRNAs can interact directly with proteins, alterations of miRNA levels through protein dysfunction or aggregation might indicate and contribute to disease activity [2]. Thus, besides providing mechanistic insights, the study of miRNAs holds potential for biomarker discovery. Circulating miRNAs in blood are exceptionally stable, easily accessible and likely reflect systemic adaptations to pathological processes. Several studies have recently demonstrated dysregulation of specific miRNAs in PD blood. However, most of these studies focus on hypothesis-driven candidates. Here, we combined an unbiased screen of serum and serum extracellular vesicles (EV)-associated miRNAs with a screen for protein binding partners of a miRNA which is highly dysregulated in PD. 

## 2. Materials and Methods

### 2.1. Ethical Approval and Study Cohort

All PD patients were diagnosed using the UK PD Society Brain Bank clinical diagnostic criteria at specialized centers for PD and were considered sporadic cases based on a negative family history. Patients with PD that fulfilled at least stage II in the Hoehn and Yahr scale were included. The Unified Parkinson’s Disease Rating Scale part III (UPDRS III) is given in Table 1. Further baseline characteristics of the different patient cohorts can be found in Table 1. All human experiments were performed in accordance with the declaration of Helsinki and approved by the Ethics Committees of Ulm University, Landesärztekammer Hessen and Munich Technical University. All study participants gave written informed consent to participate to the study. Peripheral venous blood was collected by a standardized procedure with serum collection tubes (Sarstedt, Germany). Patients and healthy controls were age- and sex-matched. For age matching, both groups were designed to have no significant difference in age when comparing average, median, and range. For sex matching, differences between investigated groups were no more than 10% in advantage of either gender.

### 2.2. Serum EV Isolation and RNA Sequencing

EVs were isolated from 9 mL serum (diluted 1:2 with PBS) by sequential centrifugation at 2000× *g* (30 min, 4 °C), 12,000× *g* (45 min, 4 °C), and 150,000× *g* (120 min, 4 °C), (further 1:2 dilution with PBS) and the EV pellet resuspended in 200 µL PBS. EV yield and purity were controlled by nanoparticle tracking (NTA) and western blot, as previously described [3]. NTA was performed with a NanoSight 2000 instrument (Malvern Instruments, Enigma Business Park, Grove Lane, UK) and confirmed single particle type with hydrodynamic diameter of 160–210 (not shown), as expected for serum EVs with this analysis [4]. EV protein was quantified by bicinchoninic acid (BCA) assay (ThermoFisher Scientific, Waltham, MA, USA) according to manufacturer’s instructions. EV markers Flotilin-1 and CD63 were detected by western blot with mouse anti-Flotillin-1 (Clone 18, BD) and rabbit anti-CD63 (H-193, Santa Cruz, CA, USA). EV miRNA was purified from EV samples with miRNeasy Kit (Qiagen, Hilden, Germany). RNA yield and quality were assessed with Fragment Analyzer (Agilent Technologies, Santa Clara, CA, USA) and miRNA pre-amplified with miScript PreAMP PCR Kit (Qiagen) due to low yield. EV miRNA was analyzed and sequenced on a HiSeq4000 platform (Illumina, San Diego, CA, USA) by the transcriptomics laboratory at the University of Göttingen.

### 2.3. Serum miRNA Analysis

Serum miRNA was analyzed as previously described [5]. All serum samples for microarray analysis were acquired in one center (Ulm University), processed according to standardized procedures and stored at −80 °C. Total RNA was extracted from 10 mL serum with QIAzol (Qiagen), precipitated with 1.4 volumes of isopropanol overnight at −20 °C, resuspended in RNAse-free water, and purified with the miRNeasy Kit (Qiagen). All array measurements were run in one batch and quality control was performed with the Agilent 2100 Bioanalyzer (Agilent Technologies) and RNA 6000 Nano Kit [6]. 200 ng of total RNA was labelled using the FlashTagTM Biotin HSR RNA Labeling Kit (Genisphere, Hatfield, PA, USA) according to the manufacturer’s protocol. The miRNAs were hybridized to AffymetrixTM miRNA 3.0 arrays, and arrays were stained and washed according to the manufacturer’s protocol on a GeneChip Fluidics Station 450 (Affymetrix, Santa Clara, CA, USA). The arrays were analyzed by the Affymetrix GeneChip Scanner 3000 and the Affymetrix Expression ConsoleTM software. Raw feature data were normalized using the RMA + DBAG algorithm and log2 intensity expression.

### 2.4. Protein Pull-Down

For pull-down of proteins with biotinylated miRNAs, naïve HepG2 cells (RRID: CVCL_0027, no entry in ICLAC) were lysed with ice-cold miRNA buffer (25 mM Tris, 150 mM KCl, 2.5 mM MgCl_2_ 0.5% NP-40, pH 7.5), the cell lysate sonicated and lipids removed from the lysate by repeated centrifugation (3× 15 min at 10,000× *g*, 4 °C) and removal of the floating phase. Cell lysate was then pre-cleared with streptavidin beads (overnight, 4 °C) and incubated with biotinylated miRNAs (Biomers, Ulm, Germany) with a final concentration of 1 µM for 2 h at 4 °C. The biotinylated miRNAs were then collected with fresh streptavidin beads (2 h, 4 °C). The beads were washed 5x with miRNA-buffer before elution of proteins for western blot. Primary antibodies against HNF4a (Biozol #BYT-ORB228944-50) and MTIF3 (Origene #TA800421) for western blot analysis were used in a dilution of 1:1000. Densitometric analysis of western blot analysis was performed with FusionCaptAdvance software from Vilber Smart Imaging. Extracted values were analyzed with a paired *t*-test.

### 2.5. Quantitative Real-Time PCR

Samples for the quantitative real-time PCRs (qRT-PCRs) were acquired in three different centers (Paracelsus-Elena-Klinik, Kassel, Ulm University, and Munich Technical University) and purified as described above. Quantitative real-time PCRs (qRT-PCRs) were carried out according to manufacturer’s protocol, using the miScript II PCR system (Qiagen). qRT-PCR data were analyzed as described before.

### 2.6. Protein Microarrays

miRNA/protein interactions were studied with the ProtoArray^®^ Human Protein Microarray v5.0 (ThermoFisher Scientific). The microarrays were equilibrated to 4 °C, blocked for 1 h (20 mM Tris-HCl pH 7.5, 1 mM DTT, 50 µg/mL yeast tRNA, 1% BSA) and 1 ng of labeled 5’-labeled (atto488 or atto633) miRNAs (Biomers) was hybridized in 1 mL of binding buffer (20 mM Tris-HCl pH 7.5, 150 mM KCl, 2.5 mM MgCl2, 1 mM DTT, 0.25 U/µL RNAseOUT, 50 µg/mL yeast tRNA) for 90 min at 4 °C. The microarrays were then washed three times with washing buffer (20 mM Tris-HCl pH 7.5, 150 mM KCl, 2.5 mM MgCl_2_, 1 mM DTT, 0.1 U/µL RNAseOUT, 50 µg/mL yeast tRNA), once with washing buffer without yeast tRNA, three times with distilled water to remove salts (RT), and dried by centrifuging at 200× *g* (1 min). Slides were scanned with a Typhoon 9400 laser scanner (GE Healthcare, Chicago, IL, USA).

### 2.7. Data Analysis

For miRNA microarray data, summary values for each probe set were calculated using ExpressionTM Console software (Build 1.3.1.187). Raw data were normalized with RMA + DBAG and log2 intensity expression. miEAA was used for miRNA annotation with miRbase v22. Further analyses were performed using BRBArrayTools v4.6.1 (http://linus.nci.nih.gov/BRB-ArrayTools.html, accessed on 19 February 2021) and R 3.6.3. Linear regression and ROC analysis of miRNAs as biomarkers was performed with SPSS and the pROC R package [7]. The volcano plot was generated with the R package manhattanly.

The statistical evaluation of the EV-miRNA sequencing data was carried out with the Oasis web platform [8]. After removing the adapter sequence and quality control, reads per sample were aligned to the human genome (hg19) and the number of reads quantified. Reads that did not match the human genome were compared with viral genomes to control for contamination with viral RNA. Matched miRNA sequences were then compared with miRbase V.22. Normalization, statistical analysis, differential expression, and cluster analyses were performed with the DESeq2 package [9]. MiRNAs with <5 reads after normalization were excluded from further analysis and a corrected *p*-value (padj) ≤0.05 set as threshold.

Pseudocolored protein microarray images were analyzed with the Protoarray prospector software (ThermoFisher Scientific). Small molecule–protein interaction analysis was used with background subtraction correction, signal scatter compensation, and Benjamini–Hochberg correction. Z-factors of all intensities [10] were calculated and a positive miRNA-protein binding event defined as z-factor >0.4. Gene enrichment and PFAM domain analysis against all proteins spotted on the microarray were performed with DAVID [11].

Statistical analysis was performed with R 3.6.3. Cluster analysis and abundance heatmaps were generated using the Genesis software [12]. Pathway and upstream regulator analyses were performed with ingenuity pathway analysis (IPA) [13].

## 3. Results

**Figure 1:**
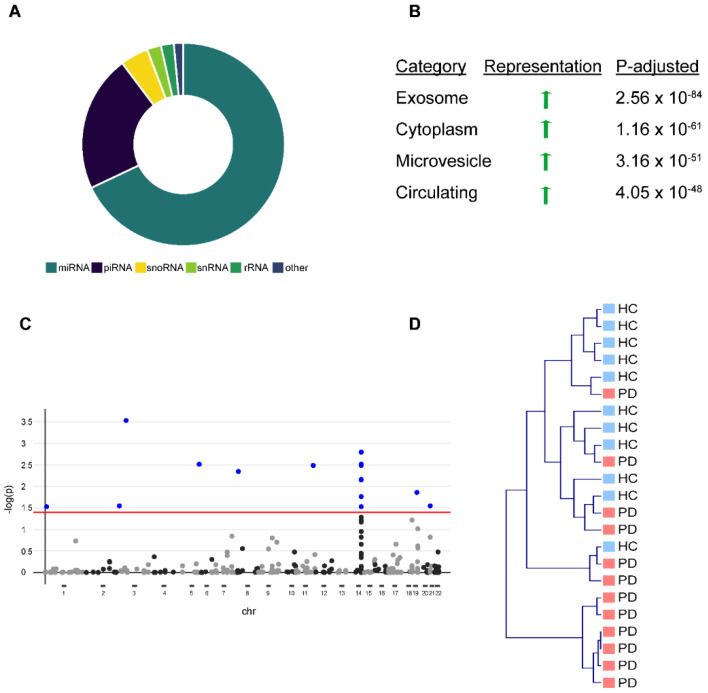
Differential expression of miRNAs in serum-derived exosomes of Parkinson‘s disease (PD) patients vs. healthy controls (HC). (**A**) Donut chart showing different non-coding RNA (ncRNA) species detected in serum-derived exosomes with the majority being miRNAs. (**B**) Functional characterization of detected miRNAs using exRNA forms (miRandola) categorization shows very strong overrepresentation of exosome, microvesicle, and circulation-associated categories. (**C**) Shows hierarchical clustering of PD patients and HC based on differential miRNA representation of exosomes with many outliers. An adequate separation between PD patients and HC is not possible. (**D**) Manhattan plot shows differential miRNA expression for every miRNA based on chromosome coordinates. Every plot represents a single miRNA. Blue-filled dots represent miRNAs with an FDR < 0.05. Red line represents a significance level of FDR < 0.05.

To obtain a comprehensive picture of the circulating miRNA profile in PD, we analyzed serum EV-associated miRNA and total serum miRNA in healthy controls and PD patients. We characterized serum EV-associated miRNAs by isolating serum EVs from 12 PD patients and 12 age/sex-matched healthy controls (Table 1) and confirmed the EVs’ purity by western blot and nanoparticle tracking as previously described [3]. We were able to detect 365 (69%) miRNAs, 117 (22%) piRNAs, 24 (5%) snoRNAs, 12 (2%) snRNAs, and 11 (2%) rRNAs in EVs with a threshold of ≥5 reads (Figure 1A), including miRNAs from a previously described miRNA-cluster on chromosome 14, which is selectively exported from cells into EVs [14]. Over-representation analysis against existing bioinformatical databases confirmed a strong overrepresentation of EV-associated terms (e.g., “exosome”, “microvesicle circulating”) (Figure 1B). After correction for multiple testing (FDR, *q* < 0.05) [15], we observed 16 miRNAs that were significantly altered in PD EVs (Figure 1C). However, unbiased hierarchical cluster analysis (complete linkage, cosine correlation) based on the abundance of all 365 miRNAs showed only a moderate strength in separating healthy controls from PD patients (Figure 1D).

**Figure 2:**
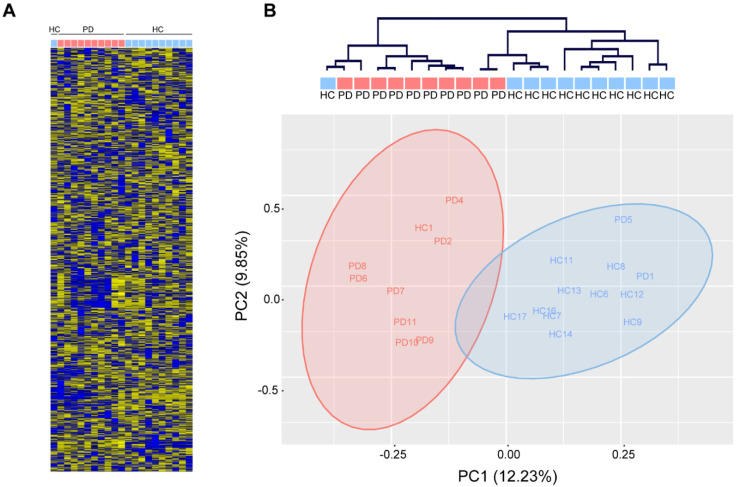
Analysis of circulating miRNAs in serum of patients with Parkinson‘s disease (PD) and healthy controls (HC). (**A**) MiRNA expression measured with miRNA arrays show distinct miRNA expression patterns in PD patients (red) and healthy controls (blue). (**B**) Unsupervised hierarchical clustering (**top**) and principal component analysis (PCA, **bottom**) show robust separation of PD patients from healthy controls (*n* = 11/10 HC/PD).

In contrast, unsupervised hierarchical clustering based on the relative abundance of 1729 mature human miRNAs (miRbase v22.0, Affymetrix miRNA 3.0) in total serum of 10 PD patients and 11 age-matched healthy controls (Table 1) robustly separated PD patients from healthy controls (Fisher’s exact: ** *p* < 0.01) and revealed a few loosely-defined clusters of co-regulated miRNAs (Figure 2A,B). Gross miRNA abundance was not biased towards up- or down-regulation in either group and was not biased by gender or age (data not shown). In addition, we did not observe a difference in hemolysis between PD and healthy control serum, which could confound the abundance of serum miRNAs (data not shown). Principal component analysis reveals a clear separation along the first principal component, which accounted for 12.23% of the variation within the data (Figure 2B).

**Figure 3:**
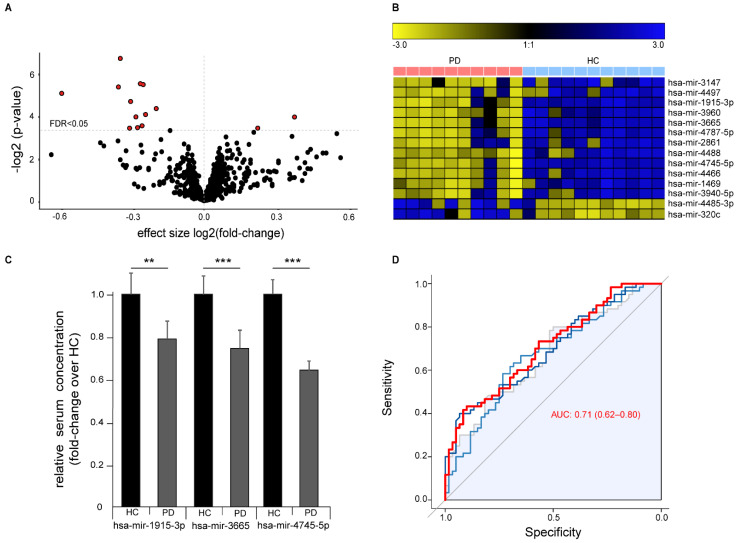
Differential expression of miRNAs in serum of Parkinson‘s disease (PD) patients versus healthy controls (HC). (**A**) Volcano plot showing negative logarithmic *p*-values versus effect size of differential miRNA expression in PD over healthy controls. Every dot represents a single miRNA (*n* = 1729), red filled dots represent miRNAs with an FDR < 0.05. Dotted horizontal line represents an FDR < 0.05. (**B**) Heatmap visualization of the expression of the 14 significantly altered miRNAs with an FDR < 0.05 (row z-score). (**C**) Validation of altered miRNA expression of top three differentially abundant miRNAs by RT-qPCR in an independent cohort from three different centers (*n* = 60/60 HC/PD, mean +/− SEM, ** *p* < 0.01, *** *p* < 0.001). (**D**) ROC plot showing diagnostic sensitivity and specificity to predict the disease status (PD) for different cutoffs. Grey represents hsa-mir-3665, light blue represents hsa-mir-1915-3p, and dark blue represents hsa-mir-4745-5p. The red line represents a logistic regression model based on all three miRNAs combined AUC (0.95 CI).

Differential abundance analysis identified 14 significantly abundant serum miRNAs (FDR *q* < 0.05, Figure 3A,B). Of these, 12 miRNAs were down-regulated and two miRNAs up-regulated in PD and included 11 miRNAs that we previously found are dysregulated in ALS 5. We validated the differential abundance of the three miRNAs with the highest difference between healthy controls and PD patients (hsa-mir-3665, hsa-mir-4745-5p, and hsa-mir-1915-3p) by RT-qPCR with an independent cohort composed of samples collected and prepared at three independent centers according to different standard operation procedures (Figure 3C). Sample size from each center was chosen to exceed 0.99 statistical power for the effect size in the discovery cohort. We were able to confirm the significant down-regulation of all three miRNAs in PD. ROC analysis with all three miRNAs showed a moderate strength in separating PD cases from controls (ROC of logistic regression analysis: AUC 0.71 (95% CI 0.62–0.80)), thus indicating only a limited value as a biomarker for PD with this method (Figure 3D).

**Figure 4:**
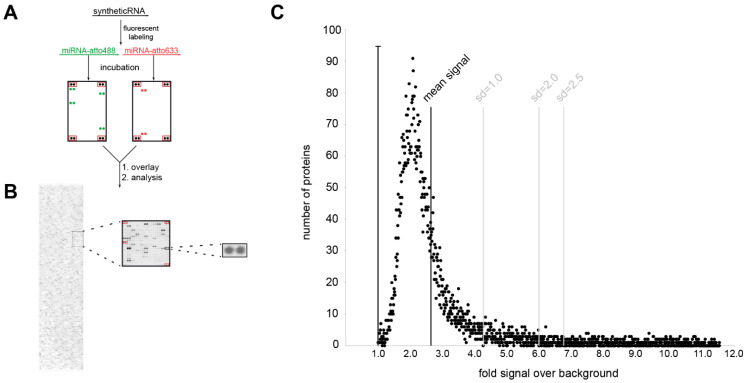
Investigation of miRNA protein binding partners with human protein microarrays. (**A**) Experimental workflow. (**B**) miRNA incubation signal on protein microarray. Signal is shown for hsa-mir4747-5p-atto488; panels on the left show the entire microarray spotted with ~9400 recombinant human proteins, the middle panel is an enlarged 484 protein spot sub-array, and the right panel is the enlarged spot for the MTIF3 protein (all proteins spotted in duplicate, sub-array positive controls boxed in red). (**C**) Shows miRNA/protein binding distribution in relation to background signal.

Next, we aimed to identify protein-binding partners of dysregulated miRNAs in PD. To this end, we performed a large-scale analysis of potential binding partners of the serum miRNA with the largest difference between PD patients and healthy controls hsa-mir-4745-5p (fold change HC vs. PD = 3.9) by incubation on protein microarrays. To distinguish between disease-specific and non-disease-related unspecific miRNA-protein binding, human protein microarrays were probed in parallel with the non-PD-regulated miRNA hsa-mir-92a-3p (*q* > 0.96, fold change HC vs. PD = 0.86) and with cel-mir-39-3p of non-human origin. The schematic workflow of this approach is depicted in Figure 4A,B. Briefly, miRNAs were synthesized, labeled with a fluorescent dye (atto488 or atto633), probed on human protein microarrays containing 9125 proteins, and scanned on a laser scanner. Laser scanning and digital image analysis showed many proteins (2–5%) with a signal intensity ratio over 1 (background), suggesting that global nonspecific fluorescence is not a major concern (Figure 4C).

**Figure 5:**
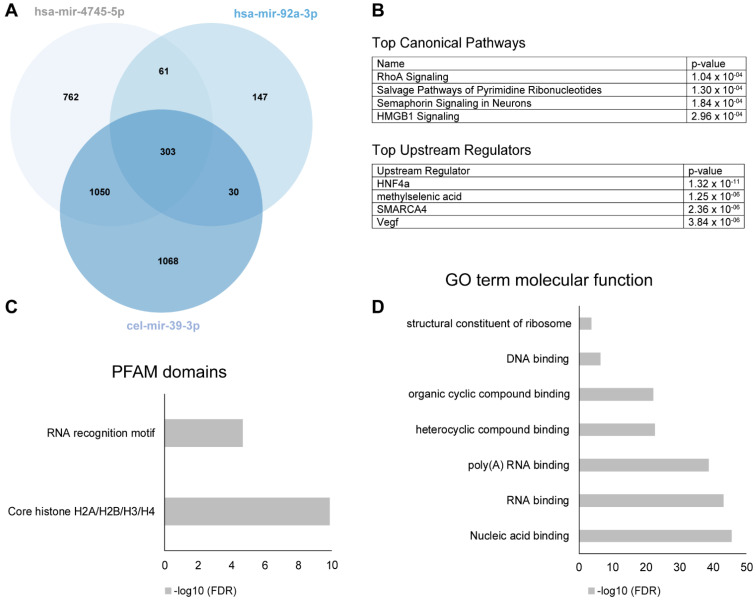
(**A**) Venn diagram of numbers of miRNA binding partners of hsa-mir-4745-5p (top regulated miRNA in PD serum), hsa-mir-92a-3p (not regulated in PD), and cel-mir-39-3p (nonhuman controls from C. elegans). (**B**) Top canonical pathways and upstream regulators of the significant protein binding partners unique for hsa-mir-4745-5p (*n* = 762) using ingenuity pathway analysis. (**C**,**D**) show top PFAM-domains and Gene Ontology (GO) term molecular functions of the significant protein binding partners unique for hsa-mir-4745-5p (*n* = 762).

The definition of a significant miRNA-protein binding event was defined by a Z-factor > 0.4 [10]. 2451 of the proteins bound to cel-mir39-3p and were considered to have only a general, non-PD-related miRNA-protein interaction (Figure 5A). 823 of the remaining proteins hybridized significantly to the PD-dysregulated hsa-mir-4745-5p, while only 208 hybridized to the non-PD dysregulated has-mir-92a-3p, including 61 proteins bound by both miRNAs. 762 protein interaction partners were unique to hsa-mir-4745-5p.

**Figure 6:**
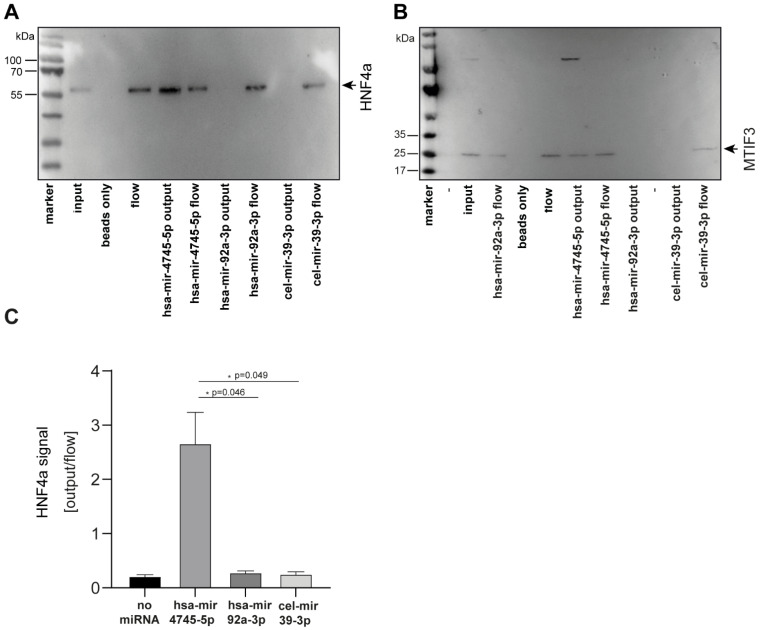
Western blot analysis confirms specific binding of hsa-mir-4745-5p with HNF4a protein (**A**) and MTIF3 protein (**B**). Non-regulated hsa-mir-92a-3p and nonhuman cel-mir-39-3p from C. elegans do not show protein interaction with HNF4a and MTIF3. (**C**) Densitometric analysis of western blots. Values are expressed as means +/− SEM, (HNF4a, *n* = 3).

To perform biochemical validation of these findings, we selected the top two candidates of the newly-identified unique hsa-mir-4745-5p interaction partners and studied miRNA protein interaction by pull-down experiments (Figure 6). First, MTIF3, which was identified as the protein-binding partner of hsa-mir-4745-5p with the highest Z-factor (0.911) in the protein microarray approach, was studied. Using biotin-labeled-hsa-mir-4745-5p, MTIF3 was pulled down from full-cell lysate, whereas the biotin-labeled-controls hsa-mir-92a-3p and cel-mir-39-3p did not pull down MTIF3, thus confirming the novel hsa-mir4745-3p-MTIF3 interaction (Figure 6A). Furthermore, pull-down verified a specific interaction between HNF4a (second-highest Z-factor) and hsa-mir-4745-5p (Figure 6B,C). Hsa-mir-92a-3p and cel-mir-39-3p did not interact with HNF4a, proving specific hsa-mir-4745-5p and HNF4a interaction.

To gain further functional insights about all hsa-mir-4745-5p binding proteins found on the protein microarray, we performed Gene Ontology (GO) analysis using the DAVID web-based knowledgebase. DAVID analyzed 11 of the GO terms relating to the significantly altered proteins and genes and generated significance value for the observed enrichment in comparison to the background protein set of the whole protein array. GO enrichment analysis showed association to nucleic acid binding (RNA and DNA), as expected (Figure 5D). Analysis of the proteins for PFAM domains revealed an expected RNA binding motif, as well as a core histone domain (Figure 5C). Pathway analysis with ingenuity pathway analysis (IPA) demonstrated that the most significantly associated pathways are related to rho-signaling, salvage of pyrimidine ribonucleotides, semaphorine-signaling in neurons, and HMGB1 signaling (Figure 5B). Upstream regulator analysis with IPA identified four top upstream regulators (HNF4a, methylselenic acid, SMARCA 4, and VEGF). Importantly, one of them, HNF4a, was also identified as a direct interaction partner on the protein array (Figure 5B).

## 4. Discussion

In this study we identified 14 significantly dysregulated miRNAs in the serum of PD patients in a discovery cohort. While the miRNA profile found by sequencing of serum EVs miRNAs did not sufficiently separate PD patients from healthy controls, the miRNA expression profile from full serum was able to distinguish the two groups in a robust manner. The three most differentially regulated miRNAs could be confirmed in an independent cohort from three different specialized centers for PD. Moreover, this study provides the first direct link between dysregulated miRNAs in PD and their corresponding protein-binding partners.

In our discovery cohort we demonstrated that 179 miRNAs are significantly dysregulated with an uncorrected *p* < 0.05, with 14 miRNAs that met the significance threshold of a corrected FDR < 0.05 for multiple testing. From the 179 dysregulated miRNAs, several have been associated with PD in previous studies [16,17,18]. However, to our knowledge none of the 14 miRNAs that passed FDR < 0.05 in our study have been listed in previous reports. In addition, there was no overlap between the dysregulated miRNAs in PD serum and miRNAs in extracellular serum vesicles. This is possibly due to their relatively low abundance in extracellular vesicles and their heterogeneity concerning their cells of origin. Interestingly, in serum there was a large overlap with dysregulated miRNA in familial ALS and presymptomatic gene carriers, suggesting a convergent pathological mechanism in these neurodegenerative diseases that is to be further elucidated.

Many differentially regulated miRNAs have been linked to PD-associated genes via mRNA interaction leading to altered gene expression. For example, hsa-mir-7 and hsa-mir-153 have been shown to regulate SNCA mRNA and the α-synuclein protein levels in mouse models of PD [19]. In contrast, little is known about the relevance of direct miRNA/protein interaction. We hypothesize that altered miRNA levels in the serum of PD patients might be secondary due to miRNA binding to proteins or protein aggregates. Strikingly, we found that there are many miRNA–protein interactions, e.g., depending on miRNA, 2%–5% of the spotted proteins showed specific miRNA binding. We could classify proteins that interact with hsa-mir-4745-5p. Most of these proteins have a well-conserved regulatory domain in common (core histone domain h2a h2b h3, and h4), underlining their function in transcriptional regulation across species. Therefore, one might speculate that direct miRNA interaction with histone proteins influences transcriptomic plasticity.

Among those proteins that solely bind to PD-dysregulated hsa-mir-4745-5p, MITF3 and HNF4a were the top hits. Interestingly, both proteins have already been associated with PD. Polymorphisms in the MTIF3 gene have been associated with PD [20,21,22] while HNF4a mRNA levels could be identified as a longitudinal PD blood biomarker [23]. Interestingly, pathway analysis of hsa-mir-4745-5p binding partners identified HNF4a again as an upstream regulator of these proteins. Thus, we speculate that hsa-mir-4745-5p can be directly regulated by HNF4a, but also indirectly by HN4Fa-dependent altered protein functions, influencing other hsa-mir-4745-5p-binding proteins. We hypothesize that the miRNA/protein interaction takes place in the cell, as we could not detect HNF4a and MTIF3 in the serum (data not shown). Hsa-mir-4745-5p has 477 predicted targets demonstrated by miR databases, so reduced levels of hsa-miR-4745-5p in PD serum will probably have a great impact on its effectors. A recent study showed that hsa-mir-4745-5p is downregulated in neurons after MPP+ stress, suggesting an involvement of hsa-mir4745-5p in the response to oxidative stress [24].

Whether hsa-miR-4745-5p effectors within cells are affected, or whether intercellular signaling is altered, needs to be elucidated. Furthermore, whether and to what extent protein function is altered by binding of miRNAs, remains to be determined. In contrast, dysregulation of miRNAs through miRNA-protein interaction might be a possible mechanism of pathogenesis. In conclusion, protein interactors of specific dysregulated miRNAs, as demonstrated in this study for PD, might give novel insights into the pathomechanisms of non-coding RNAs.

## 5. Conclusions

Specific miRNA signatures can be detected in PD patients’ whole serum, and to some extent, serum EVs. Although of limited value as a biomarker, these signatures provide new insights into molecular pathomechanisms in PD. Furthermore, we identified novel binding partners of miRNA hsa-mir-4745-5p, which could be implicated in PD pathogenesis.

## Figures and Tables

**Table 1:** Clinical characteristics of patients and controls in each cohort. N stands for number. Data are presented as mean ± standard deviation. The motor examination of Unified Parkinson’s Disease Rating Scale part III (UPDRS III) is given in points. NA means not available.

Cohort		PD	Healthy Controls	Total
Exosomes miRNA sequencing	N	12	12	24
	Female Sex N (%)	6 (50)	6 (50)	12 (50)
	Age (years)	71.1 (±6.8)	70.0 (±6.9)	70.6 (±6.7)
	UPDRS III	14 (±8.3)		
	Family history of PD (%)	0 (0)		
Serum miRNA microarray	N	10	11	21
	Female Sex N (%)	4 (40)	5 (45)	9 (43)
	Age (years)	68.9 (±6.0)	64 (±12.6)	66.1 (±10.4)
	UPDRS III	13.4 (±6.0)		
	Family history of PD (%)	0 (0)		
Serum miRNA qPCR-Munich	N	20	20	40
	Female Sex N (%)	5 (25.0)	7 (35)	12 (40)
	Age (years)	66.2 (±11.7)	63.7 (±10.2)	64.9 (±10.9)
	UPDRS III	16.4 (±6.7)		
	Family history of PD N (%)	0 (0)		
Serum miRNA qPCR-Kassel	N	20	20	40
	Female Sex N (%)	8 (40)	9 (45)	17 (43)
	Age (years)	74.4 (±10.2)	72.1 (±9.3)	73.7 (±10.0)
	UPDRS III	NA		
	Family history of PD N (%)	0 (0)		
Serum miRNA qPCR-Ulm	N	20	20	40
	Female Sex N (%)	10 (50)	10 (50)	20 (50)
	Age (years)	68.1 (±6.2)	67.2 (±4.51)	67.6 (±5.4)
	UPDRS III	15.4 (±4.8)		
	Family history of PD N (%)	0 (0)		

## Data Availability

The datasets generated during and/or analyzed during the current study are available from the corresponding author on reasonable request.
